# 

**Published:** 1889-07-27

**Authors:** 


### SPECIAL ANNOUNCEMENT.

### MISS FLORENCE NIGHTINGALE.

The next (August) monthly part of The Hospital, and the weekly issue for 27th July, will contain a biographical sketch of Miss Florence Nightingale, tracing the remarkable career of that devoted lady, and showing the effect of her labours at the present time. The article in the August number will be accompanied by a portrait of Miss Nightingale, which has been reproduced at great cost and perfection; and it will at last be possible for every member of the nursing profession to possess the likeness of one who, by a life of self-sacrifice and philanthropic labour, has raised herself to pre-eminence among women. The monthly number will be issued at sixpence as usual, but orders should be sent early.

We regret that, owing to an accident which has happened to the plate at the collotyping works where this portrait is being produced, we shall be compelled to postpone its publication to the next (September) monthly part. Though the delay thus caused is much to be regretted, there is consolation in the knowledge that a really excellent portrait of Miss Nightingale will be within reach of our readers a few weeks hence.

### NOTICE TO CORRESPONDENTS.

All MS., Letters, Books for Review, and other matters intended for the Editor, should be addressed The Editor, The Lodge, Porchester Square, London, W.

### Notice to Advertisers and Subscribers.

All Advertisements, Orders for Copies of The Hospital, and Business Communications should be addressed to The Publisher, *not to the Editor*, The Hospital, 139-140, Salisbury-court, Fleet-street, E.C.

Vol. V., now ready, 4s., post free. Cases for binding, may be had of the Publisher, at 1s. 6d. each.

### To Residents, Secretaries, and Matrons.

The Editor will be glad to have the Regulations and each Report as issued by Asylums, Hospitals, Convalescent and Nursing Institutions, as well as those of other Charities, and an early copy in duplicate of all Appeals and Festival Notices, etc., addressed to *The Editor, The Lodge, Porchester-square, London, W.* Any superintendent, secretary, matron, or other chief official who regularly sends such information may be registered, on application to the Publisher, to receive free of cost a cover for binding The Hospital in half-yearly volumes.

### Amusements and Relaxation.

### Second Quarterly Word Competition Commenced July 6th, ends September 28th.

Three Prizes of 15s., 10s., 5s., will be given for the largest number of words derived from the words set for dissection.

Proper names, abbreviations, foreign words, words of less than four letters, and repetitions are barred; plurals, and past and present participles of verbs, are allowed. Nuttall's *Standard* dictionary only to be used.

N.B.—Word dissections must be sent in WEEKLY, arranged alphabetically, with correct total affixed.

The word for dissection for this, the *Fourth* week of the quarter, being

"G L A S G O W."

**Results of Second Week.**

| Names. | July 18th. | Totals. | Names. | July 18th. | Totals. |
|---|---|---|---|---|---|
| Nurse Duty | 159 | 190 | Secnarf | 152 | 175 |
| N'importe Qui | 134 | 164 | Rover | 142 | 165 |
| Jumps | — | 30 | Leirion | 108 | 130 |
| Tinie | 156 | 186 | Matron | 66 | 87 |
| Patience | 151 | 181 | F. C. J. | 101 | 120 |
| Amicus | 153 | 183 | Percy Verance | — | 18 |
| Broxbourne | 154 | 183 | Gleniffer | 111 | 129 |
| Lauriston | 151 | 180 | Gypsye | 140 | 157 |
| Lightowlers | 154 | 183 | Ladyr | 83 | 100 |
| Speedwell | 142 | 170 | Nurse Amie | 74 | 89 |
| Sister | 146 | 174 | A. B. | 114 | 129 |
| Rattler | — | 28 | W. G. R. | 128 | 143 |
| Paignton | 105 | 132 | Essie | 123 | 133 |
| Fassifern | 114 | 141 | Cowboy Bill | 133 | 133 |
| W. R. E. | 152 | 179 | M. L. A. | 87 | 87 |
| Esperance | 154 | 180 | Little Tuppy | 77 | 77 |
| M. W. | 160 | 186 | Embryo | 56 | 56 |
| Isabel | 142 | 168 | K. Jarman | 43 | 43 |
| Buxton | 97 | 122 | Reveal | 149 | 149 |
| Nux Vom | 142 | 167 | Jenny Wren | 104 | 131 |
| Qu'appelle | 133 | 158 | Condorcet | 133 | 156 |
| Spes | — | 24 | | | |

**Notice to Correspondents.**

**First Week.—Jenny Wren, 27; Rover, 23; Condorcet, 23.**

*Cowboy Bill.*—Competitions must be sent in weekly.

N.B.—All letters referring to this page which do not arrive at 139 and 140, Salisbury-court, London, E.C., by the *first post on Thursdays*, and are not addressed PRIZE EDITOR, will in future be disqualified and disregarded

### Scraps and Gleanings.

A Leper butcher has been arrested in Bombay.

Wroughton only raised £6 this year on Hospital Sunday.

The death is announced of Dr. E. W. Lane, of Harley-street.

Miss Harris is curator of the museum at the Royal Free Hospital.

Belfast Hospital for Skin Diseases treated 1,194 patients last year.

Richmond Hospital has treated 168 in-patients in the last six months.

Sir James Crichton Brown gave the prizes at St. Mary's on July 18th.

The *British Medical Journal* has now a circulation of over 15,500 copies.

A new hospital was opened in the north of Berlin on the 1st of this month.

Stanley Park Gala in aid of the Liverpool hospitals realised over £5,000.

Norfolk and Norwich Hospital treated 338 in-patients during the last quarter.

Dr. G. B. Morgan has been appointed house surgeon to Sunderland Infirmary.

The Rev. F. W. Piercy has been elected chaplain of Bedford General Infirmary.

The Empress Frederick lately visited the consumption hospital at Falkimstein.

Dr. Michael Callan has been appointed Justice of the Peace for County Louth.

A Dictionary of Medical Specialists has been compiled by Mr. W. P. W. Phillimore.

Hydrophobia and heat prevail at Odessa. In 20 days 38 cases of hydrophobia occurred.

Croydon General Hospital has received £24 from the late collections at Sutton Parish Church.

Lady Suffield is suffering from measles, which of late has been very prevalent in the West-end.

The Clarence Barracks at Portsmouth are to be pulled down, owing to their unsanitary condition.

Dr. Edwin Goodall has been appointed assistant medical officer at West Riding Lunatic Asylum.

Dr. Humphrey J. Broomfield has been elected assistant physician to the City of Dublin Hospital.

Dr. Malet, of Wolverhampton, has been presented with an armchair on the occasion of his marriage.

Dr. Hattie Jones has been appointed assistant physician at West Virginia Hospital for the Insane.

Birmingham Eye Hospital does an astonishing work. 20,600 patients have been treated during the year.

The George Henry Lewes Studentship for original research in physiology will be filled up in October next.

Mrs. Peel was At Home in the Speaker's House on July 16th, in aid of the Children's Country Holiday Fund.

Dr. Alan Herbert, physician to the British Embassy at Paris, has been decorated with the Legion of Honour.

A house has been taken at Plumpton to act as a temporary convalescent home for poor Brighton children.

Mrs. Bramwell Booth is collecting funds to start a small hospital in connection with the work of the Salvation Army.

The *Lady's Pictorial* for last Saturday contained a bright little poem on Hospital Saturday, entitled "For Charity's Sake."

Mr. H. R. Bird lately directed a performance of Handel's "Water Music," in the new building of Cheyne Hospital for Sick Children.

The Skircoat Freeholders have handed over to the Halifax New Infirmary Committee the balance of £264 in the bank belonging to them.

The Lady Superintendent of the Nurses' Institute, Dover, says unless she receives further support her nurses can no longer gratuitously attend the sick poor.

The Lord Mayor's Fund has been brought into use to pay the expenses of a small girl lately bitten by a mad dog at Bromley. The child has been sent to Paris to the Pasteur Institute.

The committee of the Hospital for Sick Children, Great Ormond-street, Bloomsbury, have received a donation of £1,000 towards the building fund, from Mr. Junius S. Morgan.

Typhoid fever has broken out in the Marine Hospital, near Trieste, which is established for the nursing of Austrian scrofulous children, and the establishment will have to be evacuated.

Swindon Victoria Hospital has held its first annual meeting, though the wards have only been opened six months. All is working harmoniously, and the townspeople are proving generous.

The total number of lunatics, idiots, and persons of unsound mind in England and Wales on the 1st of January last was 84,340, being an increase of 1,697 on the numbers returned in the previous year.

Mr. Herbert de Stern has promised £2,000 to erect a clock tower and drinking fountain, in memory of his father, the late Baron de Stern, in front of the People's Palace and adjacent to the Mile-end-road.

The Merchant Taylors' Company have voted £31 10s. to the Lord Mayor's Fund in aid of the Pasteur Institute. Sir Henry Roscoe, M.P., has given £10; Dr. George Johnson, F.R.S., £5 5s.; Mr. Lennox Browne, £5; Mrs. Edmund Potter, £10; and Mr. C. B. Pitman, £5.

Mrs. Garrett Anderson has held a meeting at her house, at which the ladies who had assisted her in raising the £20,000 required for the building fund of the new Hospital for Women were present, and she was able to announce that the whole of that sum had been collected.

